# Marine algal natural products with anti-oxidative, anti-inflammatory, and anti-cancer properties

**DOI:** 10.1186/1475-2867-13-55

**Published:** 2013-06-03

**Authors:** Jin-Ching Lee, Ming-Feng Hou, Hurng-Wern Huang, Fang-Rong Chang, Chi-Chen Yeh, Jen-Yang Tang, Hsueh-Wei Chang

**Affiliations:** 1 Department of Biotechnology, College of Life Science Kaohsiung Medical University, Kaohsiung, Taiwan; 2 Institute of Clinical Medicine, Kaohsiung Medical University, Kaohsiung, Taiwan; 3 Kaohsiung Municipal Ta-Tung Hospital, Kaohsiung, Taiwan; 4 Cancer Center, Kaohsiung Medical University Hospital, Kaohsiung Medical University, Kaohsiung, Taiwan; 5 Institute of Biomedical Science, National Sun Yat-Sen University, Kaohsiung, Taiwan; 6 Graduate Institute of Natural Products, College of Pharmacy, Kaohsiung Medical University, Kaohsiung, Taiwan; 7 Department of Radiation Oncology, Faculty of Medicine, College of Medicine, Kaohsiung Medical University, Kaohsiung, Taiwan; 8 Department of Radiation Oncology, Kaohsiung Medical University Hospital, Kaohsiung, Taiwan; 9 Department of Biomedical Science and Environmental Biology, Kaohsiung Medical University, Kaohsiung, Taiwan

**Keywords:** Algae, ROS, Antioxidant, Inflammation, Antinociceptive, Anti-cancer

## Abstract

For their various bioactivities, biomaterials derived from marine algae are important ingredients in many products, such as cosmetics and drugs for treating cancer and other diseases. This mini-review comprehensively compares the bioactivities and biological functions of biomaterials from red, green, brown, and blue-green algae. The anti-oxidative effects and bioactivities of several different crude extracts of algae have been evaluated both *in vitro a*nd *in vivo*. Natural products derived from marine algae protect cells by modulating the effects of oxidative stress. Because oxidative stress plays important roles in inflammatory reactions and in carcinogenesis, marine algal natural products have potential for use in anti-cancer and anti-inflammatory drugs.

## Introduction

**Figure 1:**
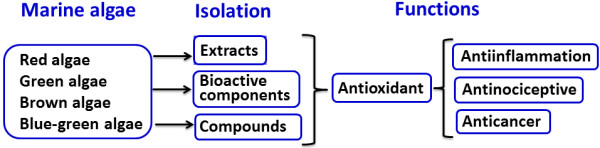
Summary of marine algal natural products with anti-oxidative, anti-inflammatory, anti-nociceptive, and anti-cancer properties.

Various bioactive compounds from marine organisms have been experimentally tested to comprehensively study the biological effects of recently developed drugs [1]. Marine algae are rich in dietary fiber, minerals, lipids, proteins, omega-3 fatty acids, essential amino acids, polysaccharides, and vitamins A, B, C, and E [2-6]. Studies on the bioactivities of marine algae have revealed numerous health-promoting effects, including anti-oxidative, anti-inflammatory, antimicrobial, and anti-cancer effects. This mini-review will evaluate the specific effects found with red (Rhodophyta) [7-11], green (Chlorophyta) [12,13], brown (Phaeophyta) [14], and blue-green [15-18] species of marine algae. Figure 1 provides a brief overview of this mini-review.

## Bioactive components of marine algae

The versatility of the functions of algae may derive from their abundant bioactive metabolites [19,20]. Well-documented bioactive metabolites of marine algae [21] include brominated phenols [20], brominated oxygen heterocyclics, nitrogen heterocyclics, kainic acids, guanidine derivatives, phenazine derivatives, amino acids and amines, sterols [22], sulfated polysaccharides [4,23,24], and prostaglandins [25]. Fucoxanthin, a type of xanthophyll and an accessory pigment in the chloroplasts of algae, has also shown various beneficial effects [26]. However, not all species of algae have health-promoting properties, as some are known to produce toxic metabolites that cause neurodegenerative disorders [27].

## Cultivation environment of marine algae

The cultivation environment may affect the bioactive metabolite content of an alga. For example, changes in cultivation conditions, such as spatial variations, can cause changes in the polyphenolic content in the *Ascophyllum nodosum* species of brown algae [28]. Additionally, in three green algae species, *Capsosiphon fulvescens*, *Enteromorpha prolifera*, and *Codium fragile,* bioactive metabolite content varies with the time of harvest [29]. Sulfated polysaccharides of the red alga *Delesseria sanguinea* (Hudson) Lamouroux can be collected throughout the year, although collection during the spring is optimal [30].

## Antioxidant activities and bioactive components of marine algae extracts

Antioxidant activities have been identified in various marine algae, including red, green, and brown algae species [31], and in their enzymatic extracts [32-34]. The antioxidant properties of extracts and bioactive components of four common marine algae are discussed here.

### Red algae

Ethanol extracts of the *Callophyllis japonica*[35] and *Gracilaria tenuistipitata*[36] species of red algae reportedly have antioxidant effects. For example, ethanol extracts of *C. japonica* suppressed H_2_O_2_-induced cellular apoptosis and activated cellular antioxidant enzymes [35]. Experiments performed with the H1299 cell line showed that treatment with an aqueous extract of *G. tenuistipitata* enhanced the recovery of these cells from H_2_O_2_-induced DNA damage, counteracts cellular proliferation, and induced G2/M arrest [36].

### Green algae

Free-radical-scavenging assays using green algae revealed antioxidant properties for the sesquiterpenoids from *Ulva fasciata* Delile [37]. *Ulva lactuca* is rich in flavonoids and has potent antioxidant properties [38]. Data obtained from animal model studies has started to shed light on the fact that the free radical scavenging effects of a hot water extract of *Ulva reticulate*[39] reduced hepatic oxidative stress.

### Brown algae

Assays of 2,2-diphenyl-1-picrylhydrazyl (DPPH)-radical scavenging have revealed antioxidant activities for the phlorotannins from the *Eisenia bicyclis*, *Ecklonia cava*, and *Ecklonia kurome* species of brown algae [40]. Methanol extracts of *Fucus vesiculosus* and *F. serratus* are known to protect Caco-2 cells from DNA damage induced by H_2_O_2_[41], but not from DNA damage induced by tert-butyl hydroperoxide [42]. Methanol extracts of *Pelvetia canaliculata* inhibited H_2_O_2_-induced superoxide dismutase depletion in Caco-2 cells [41].

### Blue-green algae

Analyses of DPPH radical scavenging activity have revealed antioxidant effects of a methanol extract of the *Anabaena* species of blue-green algae [43]. Ascorbate/iron/H_2_O_2_ assays have also revealed antioxidant effects of phycobiliprotein phycocyanin in a *Spirulina platensis* extract [44]. To date, the antinociceptive properties of blue-green algae have been less thoroughly investigated.

## Anti-inflammatory and antinociceptive properties of marine algae extracts and components

Oxidative stress plays important roles in endothelial dysfunction [45], lung disease [46], gastrointestinal dysfunction [47], and atherosclerosis [48], all of which involve inflammatory reactions. Many marine natural products that contain antioxidants are known to have anti-inflammatory effects [49-51]. Examples of the anti-inflammatory and antinociceptive properties identified in extracts and bioactive components of different marine algae are discussed here.

### Red algae

The bioactivities of the *Gracilaria* spp. of red algae have been thoroughly reviewed [52]. However, anti-inflammatory properties have been reported for only two species, *G. verrucosa* and *G. textorii*. Evidence of anti-inflammatory properties in other species of red algae is also increasing. For example, an aqueous extract of *G. tenuistipitata* suppressed virus-induced inflammation [53], a polysaccharide from *Porphyridium* sp. inhibited the replication of retroviruses [54], and an ethanol extract of *Polyopes affinis* suppressed asthmatic reactions [55]. The anti-inflammatory effects of a methanol extract of *Neorhodomela aculeata* in neurological diseases included inhibiting cellular reactive oxygen species (ROS) generation, H_2_O_2_-induced lipid peroxidation, and inducible nitric oxide synthase [56].

The anti-inflammatory effects of *Laurencia glandulifera*-derived neorogioltriol, a tricyclic brominated diterpenoid, have been demonstrated for cells that were stimulated by lipopolysaccharide (LPS) [57]. Two *Laurencia obtuse*-derived C15 acetogenins, (12Z)-cis-maneonene-D and (12E)-cis-maneonene-E, mediated the apoptosis of neutrophils during the progression of inflammatory responses [58]. A *Porphyra yezoensis* glycoprotein exhibited anti-inflammatory effects in LPS -stimulated macrophages [59]. Two enone fatty acids of *Gracilaria verrucosa,* (*E*)-10-Oxooctadec-8-enoic acid and (*E*)-9-Oxooctadec-10-enoic acid, inhibited the production of the inflammatory markers nitric oxide, TNF-α, and IL-6 [60]. Multi-mineral aquamin derived from *Lithothamnion corallioides* had anti-inflammatory effects on glial-enriched primary cultures of rat cortex [61]. Sulfated polysaccharides from *Delesseria sanguinea* (Hudson) Lamouroux also exhibited anti-inflammatory effects [30].

Some red algae species exhibit both antinociceptive and anti-inflammatory effects. For example, a methanol extract of *Bryothamnion triquetrum*[62] had both antinociceptive and anti-inflammatory properties in experiments that used Swiss mice. Antinociceptive activity was examined using an acetic acid-induced writhing test, a hot-plate test, and glutamate-/formalin-induced nociception. Anti-inflammatory effects were assessed by zymosan A-induced peritonitis analysis. Antinociceptive and anti-inflammatory activities have also been reported for a sulfated polysaccharide fraction from *Gracilaria caudate*[63], a galactan from *Gelidium crinale*[64], a mucin-binding agglutinin from *Hypnea cervicornis*[65], and a lectin from *Pterocladiella capillacea*[66].

### Green algae

Crude extracts and purified components of some green algae species are also known to have anti-inflammatory properties. For example, *Dunaliella bardawil* is rich in antioxidant beta-carotene. Studies with rats have confirmed its protective effects against acetic acid-induced small bowel inflammation [67]. Methanol extracts of *Ulva conglobata* and *U. lactuca* have shown anti-inflammatory effects in experiments that used a murine hippocampal HT22 cell line [68] and rats [69]. Studies of purified components include one on lycopene from *Chlorella marina,* which confirmed the anti-inflammatory effects of lycopene in a rat model of arthritis [70]. A sheep model of inflammation-induced cytokine production demonstrated the inhibiting effects of a mixture of phytosterols from *Dunaliella tertiolecta*[71].

Crude extracts or purified components of some green algae species reportedly had both antinociceptive and anti-inflammatory effects. For example, several nociception models have shown both antinociceptive and anti-inflammatory activities of aqueous and methanol extracts of *Caulerpa mexicana*[13,72]. Both of these activities have also been demonstrated for a lectin [73] and a sulfated polysaccharide [74] from *Caulerpa cupressoides*.

### Brown algae

A murine asthma model [75] showed that an ethanol extract of *Ecklonia cava* reduced allergic airway reactions and inflammation and inhibited LPS-induced inflammation in human endothelial cells [76]. An ethanol extract of *Ishige okamurae* also showed anti-inflammatory effects [77].

Studies of sulfated polysaccharides include animal models [78,79], which confirmed the anti-inflammatory effects of a sulfated galactofucan from *Lobophora variegata*. Alginic acid, an anionic polysaccharide in *Sargassum wightii*, exhibited anti-inflammatory effects in a rat study of adjuvant-induced arthritis [80]. Fucoidan is known to enhance the probiotic effects of Lactic acid bacteria (LAB) by immunomodulation of an anti-allergic response [81]. Fucans from *Lobophora variegata*[82], *Sargassum vulgare*[83], and *Spatoglossum schroederi*[84] also have both anti-inflammatory and antinociceptive effects.

Additionally, anti-inflammatory effects have been demonstrated for the *Myagropsis myagroides*-dervied carotenoid fucoxanthin [85], for *Eisenia bicyclis*, *Ecklonia cava-* and *Ecklonia kurome-derived* polyphenol phlorotannins [86], and for *Sargassum siliquastrum*-derived sargachromanol G [87]. Phloroglucinol, a monomer of phlorotannins that is abundant in brown algae, reportedly had an anti-oxidative stress effect and inhibited the production of inflammatory mediators in LPS-stimulated cells [88].

### Blue-green algae

Blue-green algae have well-documented protective effects against viral and bacterial infections, cancer, allergies, diabetes, inflammation, and hyperlipidemia [16]. For example, the spirulina alga had anti-oxidative and anti-inflammatory effects when assessed using a non-alcoholic steatohepatitis model [15]. C-phycocyanin [17], a biliprotein isolated from *Spirulina platensis*, suppressed inflammation by inhibiting the production of pro-inflammatory cytokines and by inhibiting the expressions of inducible nitric oxide synthase and cyclooxygeanase-2 [89]. To date, the antinociceptive properties of blue-green algae have been less thoroughly investigated.

## Anti-cancer effects of marine algae extracts and components

Because they modulate ROS generation, antioxidants have vital roles in carcinogenesis [90-93]. For example, occupational and environmental exposures to metals reportedly induce ROS generation and are associated with carcinogenesis [90]. ROS are also essential for inducing autophagy, which may be mediated by ataxia-telangiectasia mutated (ATM) and AMP activated protein kinase (AMPK) [91]. Sirtuin 3 (SIRT3), a deacetylase, is considered to be a mitochondrial fidelity protein that modulates ROS metabolism during responses to stress, such as those due to aging and carcinogenesis [92]. ROS overproduction may result in genomic instability and cellular damage, as well as carcinogenesis [94]. ROS signaling can also be induced by estrogen to increase genomic instability and promote breast cancer carcinogenesis [93].

Additionally, several drugs that may generate oxidative stress and trigger the expressions of several miRNAs and DNA damage responses have been reviewed [95,96]. For example, cells that were treated with ferric nitrilotriacetate could generate ROS and induce the overexpression of miRNA-34a. In contrast, miRNA-34a downregulation by a small interfering RNA may have inhibited the proliferation of HeLa and MCF7 cancer cells [97]. These results suggest that ROS and miRNAs are also involved in carcinogenesis.

Inflammation also has molecular links to carcinogenesis [98,99]. Therefore, pro-oxidant natural products are commonly chosen when developing anti-cancer drugs [100-102]. Examples of the anti-cancer properties of extracts and bioactive components of four marine algae are discussed here.

### Red algae

Aqueous extracts of *Gracilaria corticata*[103] and *Sargassum oligocystum*[104] inhibited the proliferation of human leukemic cell lines. Both ethanol [11] and methanol [105] extracts of *Gracilaria tenuistipitata* reportedly had anti-proliferative effects on Ca9-22 oral cancer cells and were involved in cellular apoptosis, DNA damage, and oxidative stress. Similarly, caspase-dependent apoptosis induced by a methanol extract of *Plocamium telfairiae* has been demonstrated using HT-29 colon cancer cells [106].

### Green algae

Among green algae, a hot water extract of *Capsosiphon fulvescens* that contained polysaccharides induced the apoptosis of gastric cancer cells [107] via the PI3K/Akt pathway [108,109]. Dimethylsulfoniopropionate, a tertiary sulfonium metabolite found in green algae and other algae species, exhibited anti-cancer effects in mice with Ehrlich ascites carcinoma [110].

### Brown algae

Studies of brown algae have shown that glycoproteins from *Laminaria japonica*[111] and fucoidans from *Sargassum hornery*, *Eclonia cava*, and *Costaria costata*[112] had anti-cancer effects on human colon cancer cells. Heterofucans from *Sargassum filipendula* exhibited anti-proliferative effects on cervical, prostate, and liver cancer cells [113]. A carotenoid fucoxanthin cold inhibit the growth of LNCap prostate cancer cells by arresting these cells in the G1 phase via the GDD45A and SAPK/JNK pathways [114].

### Blue-green algae

Studies of blue-green algae have confirmed the anti-cancer effects of Spirulina preparations [115], recombinant glycoproteins, specifically microcystis viridis lectin (MVL) [18], and cryptophycin [116,117].

## Conclusion

Marine algal natural products are rich sources of antioxidants. In fact, some marine algae are edible. The details for the fractionation of crude extracts described in this study showed that these extracts and their bioactive components had strong modulating effects on oxidative stress and on oxidative stress-related diseases and cancers. The antioxidant properties of several kinds of algae have been investigated for their anti-inflammatory, antinociceptive, and anti-cancer effects. In the future, these marine algae-derived materials/compounds will be used more often in pre-clinical studies for drug discovery.

## Competing interests

The authors declare that they have no competing interests.

## Authors’ contributions

J-CL did the literature search, integrated different points, and drafted the manuscript. M-FH, H-WH, F-RC, and C-CY conceived the idea and did literature search on specific points. J-YT and H-WC were involved in discussion and editing the manuscript. All authors read and approved the final manuscript.
